# Biodiversity, Survival, and Influence on Quality of Yeast Biota in Virgin Olive Oil

**DOI:** 10.3390/foods15203631

**Published:** 2026-10-13

**Authors:** Biagi Angelo Zullo

**Affiliations:** Department of Agricultural, Environmental and Food Sciences, University of Molise, Via De Sanctis, 86100 Campobasso, Italy; biagi.zullo@unimol.it

**Keywords:** extra virgin olive oil, yeast ecology, lipolysis, debittering, oil processing

## Abstract

The microbial stability and sensory quality of extra virgin olive oil (EVOO) depend largely on its oil-borne yeast biota during storage. Virgin olive oil (VOO) harbors a low-abundance yeast biota in its aqueous microphase, whereas bacteria and molds are generally inhibited by low water activity and high phenol content. The genera *Candida* (6 species), *Yamadazyma* (5 species), *Brettanomyces* (2 species), *Kuraishia* (2 species), *Nakazawaea* (2 species), and *Ogataea* (2 species) dominate, representing 70% of reported yeast diversity. Yeast survival is strongly shaped by water content, oxygen, and phenolic concentration, with populations declining over time unless free water is available. Yeast metabolic activity can be detrimental or beneficial: lipolytic species (e.g., *Candida adriatica*, *Yamadazyma terventina*) increase free acidity and oxidative susceptibility, causing sensory defects, whereas oleuropeinolytic yeasts reduce bitterness via β-glucosidase and esterase-mediated hydrolysis of oleuropein. Since additives and starter cultures are prohibited by EU regulations, controlling process parameters (like blending and decantation) remains the only sound strategy. As demonstrated in trials with Don Carlo and Coratina cultivars, selective management of endogenous yeasts successfully mitigates bitterness while keeping quality parameters within EVOO legal limits, thus balancing sensory profile, shelf life, and commercial value.

## 1. Introduction

Virgin olive oil (VOO) is obtained from the mechanical extraction of olive fruit and can be consumed without subsequent refining processes. VOO is the main dietary fat in Mediterranean countries. Interest in this type of vegetable oil arises from its particular chemical composition, characterized by the presence of antioxidant compounds and a high content of unsaturated fatty acids [1]. Freshly produced VOO appears cloudy due to the presence of microdroplets of vegetation water and solid particles of olive pulp and skin covered by a water film, which represent the suspended fraction of VOO [2,3]. During storage of VOO, the suspended fraction settles to the bottom of the container to form a sediment, and the oil appears clear. In some cases, VOO remains veiled during storage, and the factors underlying this phenomenon remain poorly understood. Industries needing to market the oil produced in the shortest time possible resort to filtration with cotton filters and cellulose filter presses before bottling. However, filtration of freshly produced VOO is controversial, as studies have shown that filtration reduces phenolic content [4] and yeast load [5], with contrasting effects on oxidative and microbial stability. Other studies, however, have demonstrated that the elimination of the sediment improves the shelf life of VOO and prevents the development of defects during storage [6]. The chemical composition of VOO is made up of 98% saponifiable fraction and 2% unsaponifiable fraction by total oil weight. The latter fraction is represented by over 230 chemical compounds, such as aliphatic and triterpenic alcohols, phytosterols, hydrocarbons, volatile compounds, and antioxidants. The major antioxidant compounds present in VOO are represented by carotenoids, tocopherols, and phenols; the latter occur at significantly higher concentrations in VOO than in other vegetable oils [7]. The polar phenolic compounds present in VOO include phenolic acids, phenolic alcohols, secoiridoids, lignans, and flavonoids, which exhibit antioxidant, anti-inflammatory, anticancer, antimicrobial, and antiviral activities [8]. During storage, VOO is subject to quality deterioration as hydrolysis, oxidation, and polymerization reactions occur in its chemical components [9,10]. Ciafardini and Zullo [11] demonstrated, for the first time, that freshly produced VOO harbors a biotic fraction represented by the microbiota which during oil extraction in the mill, colonizes the suspended material of the oil. The microbiota is made up of yeasts, bacteria, and molds. Studies conducted on the VOO microbiota have demonstrated that yeasts are able to influence the physicochemical, sensorial, and nutritional and health-promoting properties of VOO during storage [12,13]. While a previous comprehensive review by Zullo and Ciafardini [8] established the foundational framework for understanding the VOO microbiota, significant paradigm shifts have occurred over the last six years. This updated manuscript goes beyond the taxonomic classification of that era to critically examine newly identified species, such as *Novakomyces olei*, *Rasporella oleae*, and *Yamadazyma oleae*, which redefine the known yeast biodiversity of VOO. Furthermore, recent advancements in downstream processing—specifically the different impacts of modern industrial filtration methods and selective natural decantation—are evaluated here through a biotechnological lens, offering novel solutions for the commercial production of highly bitter extra virgin olive oils (EVOOs) without chemical additives. In the end, new evidence regarding how phenolic profiles regulate yeast dynamics was reported.

## 2. Microbiota of VOO

The oily fraction of healthy olives still attached to the tree is free of microorganisms. During oil extraction in the mill, the oily fraction is colonized by microorganisms from different sources, including the mill equipment and the olive carposphere [14,15]. The microbiota of freshly produced VOO comprises yeasts, bacteria, and molds. However, during VOO storage, bacteria and molds often die after a short time due to the low water activity and high phenolic content, while yeasts survive and constitute the dominant microbiota during storage [14,15,16].

### 2.1. Yeast Biodiversity and Distribution

The current knowledge on olive oil yeast biota comprises 27 yeast species belonging to 14 genera, all identified by D1/D2 26S rDNA sequencing (Table 1).

The genera *Candida* (6 species), *Yamadazyma* (5 species), *Brettanomyces* (2 species), *Kuraishia* (2 species), *Nakazawaea* (2 species), and *Ogataea* (2 species) dominate, representing 70% of reported yeast diversity. Remarkably, 10 of these species have been described as new species from olive oil environments, including *Brettanomyces acidodurans*, *Candida adriatica*, *Kuraishia mediterranea*, *Nakazawaea molendinolei*, *Novakomyces olei*, *Ogataea histrianica*, *Ogataea kolombanensis*, *Rasporella oleae*, *Yamadazyma oleae*, and *Yamadazyma terventina*, suggesting that VOO represents a selective ecological niche that may drive speciation. 37% of the yeast species isolated so far from VOO samples are new. Geographically, the highest biodiversity is documented in Italy with 23 species, followed by Slovenia and Spain, a pattern that likely reflects both intensive research effort and the diversity of olive cultivars and milling practices. The by-products, together with VOO, constitute the main isolation sources, although several species are also recovered from spoiled oil and decanter oil (Table 1). Among spoilage yeasts, *B. acidodurans* is a new species particularly relevant due to its acid tolerance and ability to grow at the oil-water interface, where it produces 4-ethylphenol from hydroxycinnamic acids and releases extracellular lipases that increase free acidity. These activities contribute to the “phenolic-medicinal”, “winey-vinegary”, and “fusty/muddy” sensory defects recognized by the International Olive Council (IOC) in spoiled olive oil [17,29]. Yeast species such as *Yamadazyma diddensiae* (syn. *Candida diddensiae*) and *Nakazawaea wickerhamii* (syn. *Candida wickerhamii*) have been found in commercial VOO, available in supermarkets, and VOO produced from the Moraiolo and Frantoio cultivars [13]. *Candida norvegica*, *Candida oleophila*, *Debaryomyces hansenii*, *Y. diddensiae*, and *Wickerhamomyces anomalus* were found in the newly produced Taggiasca olive oil, while only *W. anomalus* survived after six months of storage [15]. Other species including *Groenewaldozyma auringiensis*, *Lachancea fermentati*, and *N. olei* were isolated from VOO produced in Spain [17,24]. *L. fermentati* has also been found in Italy in olive oil must [22]. The yeast *Kuraishia capsulata* was isolated by us for the first time in a blended VOO, prepared from an oil of an Italian origin and one from Portugal [5]. *K. capsulata* is a generalist yeast previously isolated from soil and bark beetle galleries [30], suggesting that its presence in VOO may derive from insect vectors or environmental contamination during olive processing rather than from a specialized adaptation to the oil matrix. *C. adriatica*, *N. molendinolei*, and *Y. terventina* are yeast species that are frequently isolated from Italian oils [13,18,22]. Four new yeast species were found in sediment olive oil and named *K. mediterranea*, *O. histrianica*, *O. kolombanensis*, and *R. oleae*. The first three yeast species can assimilate methanol, produced as a byproduct of pectin metabolism, because they are pectinolytic [21,25]. Pectinolytic yeasts produce a polygalacturonase to utilize pectin for growth and have been previously identified as the causative agents of spoilage defects in table olives [31,32]. Other yeast species, including *Candida dendronema*, *Candida temnochilae*, *Saccharomyces cerevisiae*, *Yamadazyma mexicana*, and *Yamadazyma nakazawaea* were isolated from Sardinian olive oils [19]. Recently, a new species *Y. oleae* was isolated from Italian oil produced in northern Italy [27].

### 2.2. Survival of Yeasts in VOO

**Figure 1:**
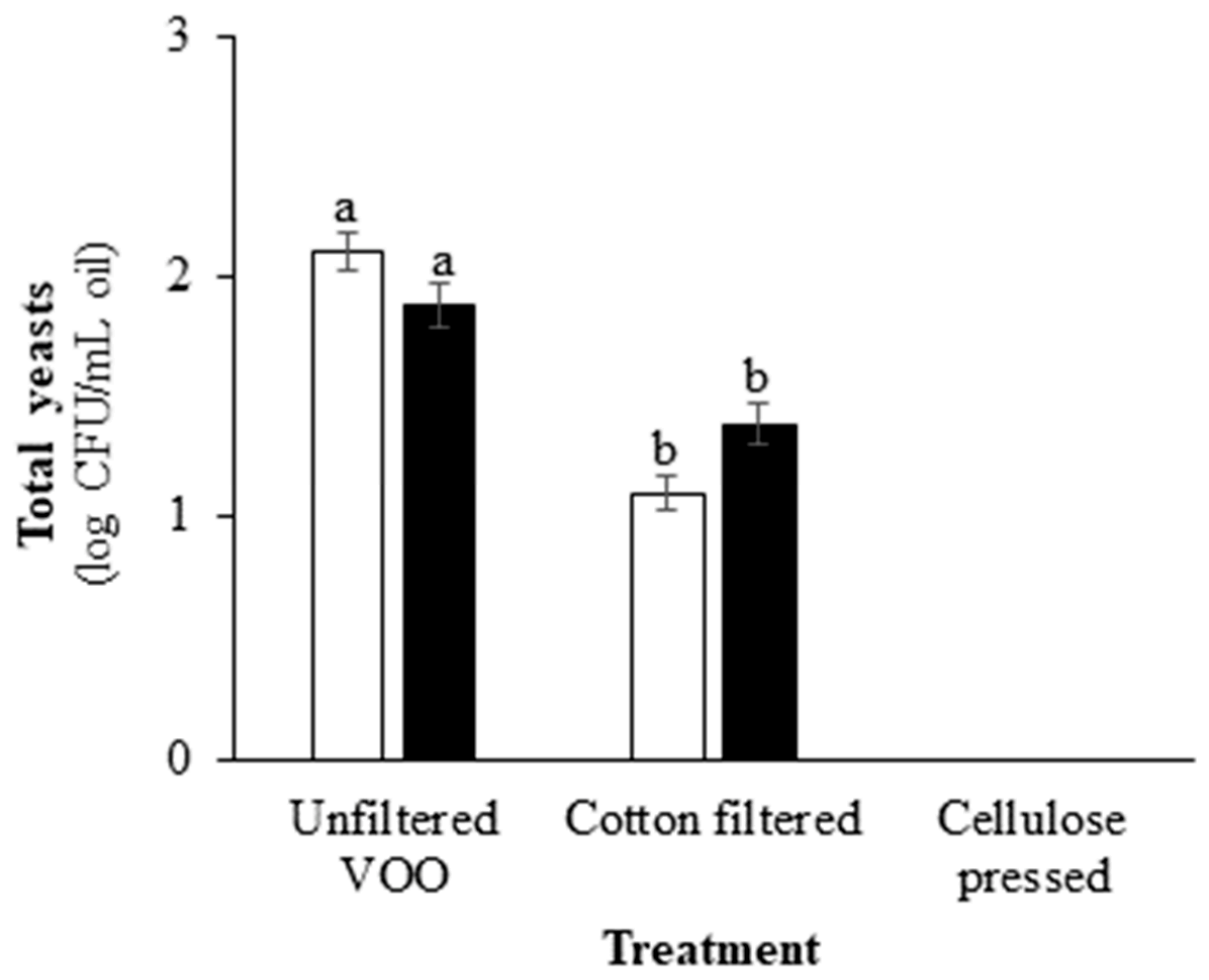
Survival of yeasts in two monocultivar virgin olive oils (VOOs) subjected to different industrial filtration methods immediately after mechanical extraction and stored for one month. Cultivars evaluated: Coratina (□) and Nera di Colletorto (■). No viable yeasts were detected in the cellulose pressed treatment after one month of storage due to the low water content of 0.06%. Error bars represent the standard deviation (*n* = 3). Different letters indicate statistically significant differences with each respective cultivar according to Duncan’s post-hoc test (*p* < 0.05). Figure adapted from Zullo et al. [5].

The concentration of yeasts present in VOO depends on several factors, such as filtration, sedimentation, and the physicochemical composition. In general, filtration reduces the initial microbial load. However, in some cases, the initial yeast concentration can be partially or completely restored by cell replication during storage. In fact, microbiological analysis of some commercially available VOO samples that appeared very clear, probably after filtration, showed low species diversity and yeast counts ranging from 3.50 to 4.20 log CFU/mL oil [23]. Zullo et al. [5] studied in depth the influence of filtration performed with cotton filters and a cellulose filter press on the biotic and abiotic components of two freshly produced monocultivar VOOs (Coratina and Nera di Colletorto). As illustrated in Figure 1 (adapted from Zullo et al. [5]), the filtration process significantly decreased the initial yeast concentration. In unfiltered control samples, the initial yeast counts were around 2 log CFU/mL. Filtration with cotton filters reduced the yeast load to levels between 1.0 and 1.4 log CFU/mL depending on the cultivar, while the use of a cellulose filter press led to a more drastic reduction. Notably, the viable yeasts that passed through the oil during filtration with the cellulose filter press, unlike those in other samples, were completely unable to survive after one month of storage, likely because the water content in the filtered oil was too low, at 0.06% (Figure 1).

In unfiltered VOOs, yeast counts were between 4.56 log CFU/mL in the newly produced and 3.03 log CFU/mL after one year of storage [33]. Yeast survival in the oily mass depends on water content, water activity, and the chemical composition of the VOO [34,35]. The water content in VOO varies from 0.15% to 0.36% (*w*/*w*), depending on the type of oil extraction system and the filtration, corresponding to a water activity of approximately 0.95–0.98. However, a water content above 0.25% (*w*/*w*) not only favors the growth of microorganisms but at the same time activates enzymes harmful to the quality of VOO [36]. A high content of phenolic compounds reduces yeast survival in VOO. In previous research, it was observed that a high phenolic content shortened the survival in VOO of some pathogenic opportunistic yeasts, including *Candida parapsilosis* [37]. Another study performed to evaluate the survival of 17 yeast strains in two monocultivar VOOs (Leccino and Coratina cultivars), characterized by different phenolic contents, after 6 months of storage, showed that the level of yeast survival was strain and phenolic content-dependent. In Coratina VOO, which is characterized by a high phenolic content, the number of yeast CFUs recovered was lower than that in Leccino VOO with the lowest phenolic content [22]. Other components of VOO, such as fatty acids and triglycerides, can also inhibit yeast growth. Laboratory trials indicated that dimorphic yeasts isolated from olive oil are sensitive to oleic acid, linoleic acid and triolein, which significantly reduced yeast growth, whereas a less inhibitory effect was observed with tricaprilin. This inhibition is attributed to the disruption of the yeast cell membrane by free fatty acids [23,38]. The blending of monovarietal VOOs represents another critical factor regulating yeast survival during storage. Research by Zullo and Ciafardini [39] revealed a more pronounced decline in viable yeast counts within blended formulations than in their single-cultivar precursors. Furthermore, basidiomycetous genera such as *Cryptococcus*, *Rhodotorula*, and *Sporobolus*, which typically inhabit the olive carposhere and foliage, fail to persist within the oil matrix during subsequent storage phases [40].

### 2.3. Bacteria and Molds

Investigation into the bacterial fraction by Pizzolante et al. [41] identified taxa including *Stenotrophomonas rhizophila*, *Pseudomonas cedrina*, *Pseudomonas stutzeri*, and *Pantoea septica* in blended oils stored for one year, derived from cultivars such as Leccino, Coratina, Ogliarola, Frantoio, and Cellina di Nardò. The persistence of certain *P. septica* strains within the oil is facilitated by their synthesis of carotenoids and bioemulsifiers. Following enrichment cultivation, Fancello et al. [42] recovered additional bacterial groups, including *Bacillus* spp., *Brevibacillus* spp., *Micrococcus* spp., *Staphylococcus* spp., *Kocuria* spp., *Lysinbacillus* spp., and various lactic acid bacteria (LAB) from diverse Italian monovarietal oils. Phenolic concentration also dictates bacterial viability; Zullo et al. [16] established that coliforms can persist and proliferate specifically in low-polyphenol environments. This was supported by challenge tests where *Escherichia coli* from the olive carposhere was fully inactivated within 15 days in high-phenolic oils, but multiplied rapidly in low-phenolic matrices. Consequently, the current literature heavily emphasizes bacterial biodiversity and its prospective biotechnological exploitation, leaving their precise impact on oil quality parameters during storage relatively underexplored. The mold content in VOO obtained from healthy olives is low, and the few molds present typically die during storage. Molds isolated from different VOO samples mostly belong to the *Aspergillus* genus [43]. Although molds are rarely detected, species of *Aspergillus* and *Penicillium* are known to produce extracellular lipolytic enzymes that can hydrolyze triacylglycerols in olive oil [44].

## 3. Influence of Yeasts on EVOO Quality

The role of oil-borne yeasts in EVOO during storage is dual, depending on the yeast species and the physicochemical composition of the oil. As summarized in Table 2, yeasts lead to both quality deterioration and improvement of EVOO. Negative effects are mainly related to the development of sensory defects and chemical changes. The inoculation of Leccino EVOO with six oil-borne yeast strains belonging to *C. adriatica*, *C. diddensiae*, and *N. wickerhamii*, after four months of storage, showed the appearance of sensory defects, such as “muddy-sediment”, “rancid” in olive oil inoculated with *C. adriatica* strain 1933, *N. wickerhamii* strain 1885, and *C. diddensiae* strains 1912 and 1913. In contrast, olive oil inoculated with *C. diddensiae* strains 1918 and 1922 was defect-free and remained in the EVOO class [34]. These results demonstrate that the appearance of sensory defects in olive oil during storage is strain-dependent. Experimental inoculation with specific indigenous yeast strains (*N. wickerhamii*, *N. molendinolei*, and *Y. terventina*) led to a significant accumulation of off-flavor volatile markers, driving their concentrations well within or above their typical analytical quantification ranges: 0.05–10.00 mg/kg for *trans*-2-heptenal, 0.20–25.00 mg/kg for 6-methyl-5-hepten-2-one, and 0.05–10.00 mg/kg for 2-octanone. Specifically, in microbiologically spoiled oils, 6-methyl-5-hepten-2-one can accumulate to average levels ranging from 0.85 mg/kg up to 7.80 mg/kg, while *trans*-2-heptenal frequently accumulates past its low odor threshold (0.042 mg/kg). Concurrently, a significant reduction in the C6 volatile carbonyl compounds associated with desirable organoleptic attributes was observed [13]. Furthermore, certain yeast taxa synthesize extracellular lipases (glycerol-ester hydrolase, EC 3.1.1.3) during storage. These enzymes cleave the acyl ester bonds of triacylglycerols, releasing free fatty acids that fundamentally lower oxidative stability and accelerate autoxidation processes [8]. The lipolytic activity of yeasts is influenced by the ratio of the aqueous and organic phases and by the content of total polar phenols. Inoculation tests of oil with lipolytic yeasts have shown that lipolytic activity reaches its maximum value when the water added to the oil is 1% for *C. adriatica* and 0.25% for *C. parapsilosis* [12]. Other laboratory experiments conducted with olive oil with increasing content of total polar phenols (89, 159, and 540 mg CAE per kg) showed that the percentage of the lipase-producing yeasts able to hydrolyze the triglycerides was respectively 100%, 67%, and 11% [45]. The bitter taste of freshly produced VOO is due to the presence of a glucoside called oleuropein, the content of which in the oil is linked to the degree of ripeness of the olives from which the oil is extracted and the cultivar [46]. During storage, the bitterness of the oil is reduced thanks to the action of some yeasts, associated with the material suspended in the oily fraction, which, through the production of β-glucosidase and esterase, hydrolyze oleuropein and its derivative oleuropein aglycons into elenolic acid, hydroxytyrosol, and glucose, which have a non-bitter taste [11]. Yeasts capable of hydrolyzing oleuropein are called oleuropeinolytic.

### Critical Evaluation of Biotic-Abiotic Interactions

**Figure 2:**
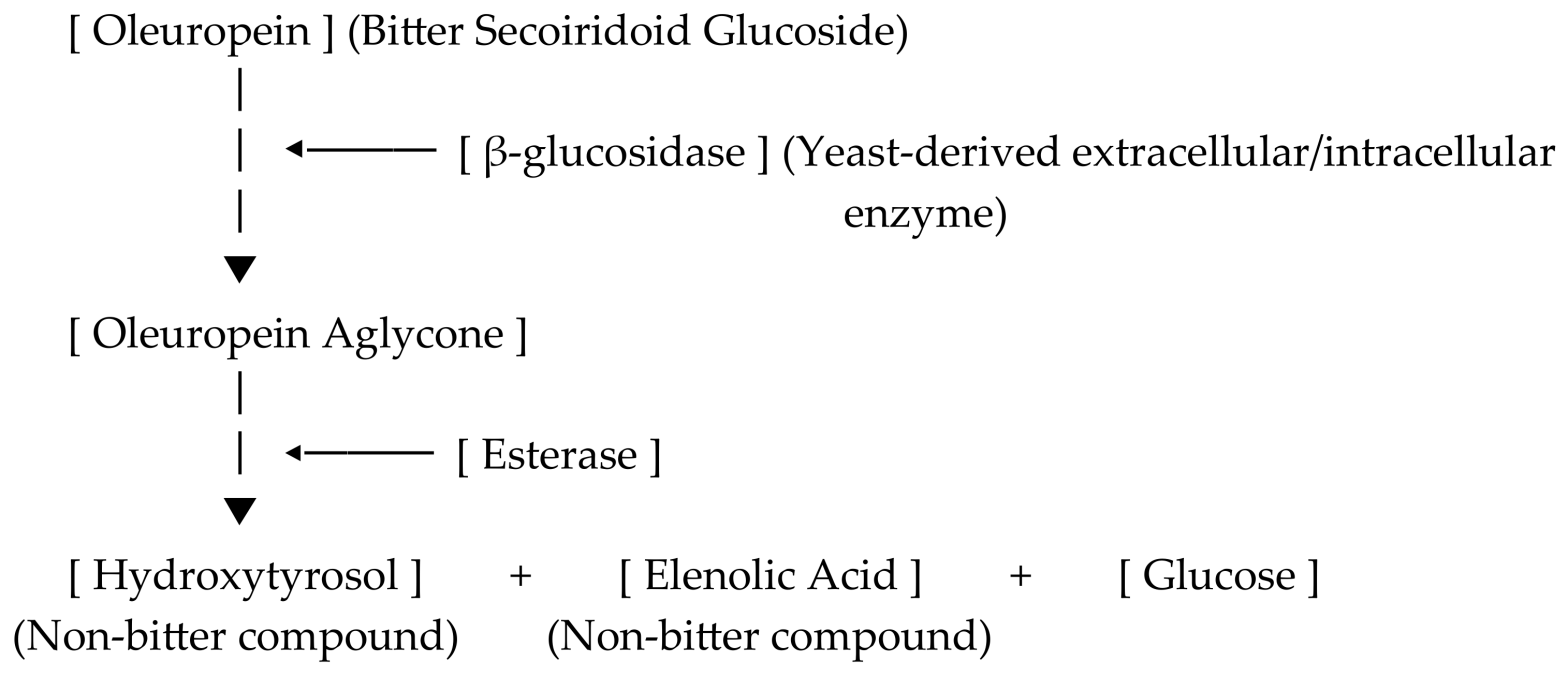
Schematic representation of the enzymatic pathway involved in the biological debittering of virgin olive oil. Endogenous oleuropeinolytic yeasts release β-glucosidase and esterase enzymes to hydrolyze the bitter secoiridoid oleuropein into non-bitter derivatives (hydroxytyrosol, elenolic acid, and glucose).

A critical analysis of the available literature reveals a complex, and sometimes contradictory, relationship between the abiotic matrix of VOO and yeast metabolic behaviour. For instance, while high phenolic concentrations are universally recognized as antimicrobial barriers that reduce overall yeast survival, they simultaneously exert a rigorous selective pressure that favors specific robust strains [22,37]. This phenomenon implies that high-polyphenol oils do not become sterile. Instead, they harbour highly specialized oleuropeinolytic or lipolytic communities. The metabolic activity of these robust strains can be triggered by minor changes in storage conditions. From an ecological perspective, this selection suggests a co-evolutionary adaptation where certain indigenous strains utilize secondary metabolites as carbon sources or possess advanced cellular detoxification systems. For example, oleuropeinolytic yeasts actively produce β-glucosidases and esterases to hydrolyze the bitter glucoside oleuropein into non-bitter derivatives like hydroxytyrosol, elenolic acid, and glucose (Figure 2).

This enzymatic pathway not only drives the natural debittering of the oil but also modulates the chemical microenvironment, as the released hydroxytyrosol remains dissolved within the organic phase, further preserving the oxidative stability and extending the chemical shelf-life of the matrix [11]. Moreover, the duality of yeast action represents a complex challenge, as the same matrix can support both beneficial debittering and detrimental lipolysis. This suggests that the organic-to-aqueous phase ratio is a hyper-critical vector regulating microbial expression. Freshly extracted, unfiltered VOO exists as a transient emulsion where microdroplets of vegetation water and solid particles are suspended within the continuous triacylglycerol matrix. This aqueous microphase concentrates both the polar phenolic compounds and the dissolved nutrients required for yeast colonization and proliferation. However, the physical stability of this emulsion changes drastically over time due to natural decantation and coalescence, leading to the formation of a bottom sediment layer [33]. As the water content shifts, localized fluctuations in water activity (aw) occur, setting off opposite biochemical pathways. While a water content above 0.25% (*w*/*w*) provides the thermodynamic baseline necessary for yeast cell replication, it also activates enzymatic pathways that can be highly harmful to the quality of VOO [8]. Lipolytic activities mediated by species like *Candida adriatica* or *Yamadazyma terventina* cleave the acyl ester bonds of triacylglycerols, releasing free fatty acids (FFAs) into the immediate microenvironment [45]. From a lipid chemistry perspective, the accumulation of these FFAs does not merely increase the titratable acidity; it fundamentally alters the autoxidation kinetics of the virgin olive oil matrix. FFAs act as potent pro-oxidants due to their amphiphilic nature [47,48]. The hydrophilic carboxylic group of FFAs preferentially aligns at the organic-aqueous interface of the oil microemulsion, the exact spatial niche where endogenous yeasts and suspended vegetation water reside [12,13]. At this interface, FFAs accelerate the initiation and propagation steps of radical chain reactions with the formation of hydroperoxides (primary oxidation products) [47,48]. Subsequently, through the β-scission reactions, the non-volatile primary hydroperoxides are transformed into low-molecular-weight, volatile secondary oxidation products, predominantly straight-chain saturated and unsaturated aldehydes (e.g., hexanal, *trans*-2-heptenal) and ketones (e.g., 2-octanone) [47,49]. These molecules are characterized by exceptionally low odor thresholds and are the direct chemical vectors behind the “rancid” and “muddy-sediment” sensory defects recognized by the IOC [13,29]. Consequently, the presence of highly lipolytic yeast strains induces a distinct dynamic evolution in classical chemical oxidation indices during storage [22,49]. The peroxide value (PV), which reflects the instantaneous concentration of primary hydroperoxide, exhibits a non-linear trend; it may initially plateau or even decline in heavily contaminated oil, as the rate of hydroperoxide decomposition mediated by FFAs outpaces their formation [47,49]. This makes PV an unreliable standalone indicator for microbially degraded oils. Conversely, spectrophotometric ultra-violet absorbances provide a more reliable kinetic trace. The k_232_ value, which selectively measures the conjugated dienes formed during the initial rearrangement of hydroperoxides, tracks the early phases of radical propagation [47]. Concurrently, the k_270_ value, measuring conjugated trienes and secondary carbonyl compounds, shows a progressive and sharp upward trajectory [49]. Under active microbial spoilage, the acceleration of the β-scission pathway causes k_270_ to breach the strict statutory limits established for the extra virgin olive oil class long before the primary autoxidation markers suggest severe degradation [49,50]. Paradoxically, these accumulated FFAs act as potent amphiphilic surfactants that disrupt yeast cell membranes, leading to intracellular damage and a subsequent decline in viable cell counts [23,38]. This creates a self-limiting but highly destructive kinetic loop where enzymatic spoilage directly compromises the survival of its own biological source. Current scientific consensus heavily relies on static laboratory inoculation trials; however, under dynamic industrial storage conditions, these interactions become far less predictable. In large-scale industrial stainless steel silos, abiotic parameters like micro-oxygenation, vertical temperature gradients, and localized water activity fluctuations dramatically alter the phenotypic expression of the resident yeast biota, frequently shifting their operational role from quality-improving biocatalysts to active spoilage agents. The formation of a bottom sediment layer during bulk storage concentrates cells at the oil-water interface, creating localized pockets that can trigger detrimental metabolic shifts. Under these conditions, generalist or spoilage species, such as *Brettanomyces acidodurans*, may outcompete specialized strains, accelerating the synthesis of off-flavor volatile markers like 4-ethylphenol, acetic acid, and short-chain fatty acids at the expense of desirable volatile compounds (Table 2) [17,29]. Consequently, future industrial applications cannot treat the VOO matrix as a static system. Understanding the kinetic cross-talk between minor polar components, moisture content, and physical storage architecture is paramount. This knowledge is essential to successfully manage endogenous microbial populations while maintaining the commercial and regulatory thresholds of the extra virgin olive oil class.

## 4. Biotechnologies for Debittering Highly Bitter EVOO

Certain monovarietal extra virgin olive oils (EVOOs), particularly those extracted from specific cultivars such as Don Carlo and Coratina, frequently exhibit an intense, aggressive bitter note immediately following mechanical extraction. This sensory sharpness is directly correlated with an exceptionally high concentration of polar phenolic compounds, predominantly secoiridoids like oleuropein, demethyloleuropein, and their respective aglycone derivatives. While these high polyphenol profiles are highly desirable from a technological and nutritional standpoint, conferring superior oxidative stability, extended shelf-life, and robust health-promoting properties [1], the resulting bitterness often exceeds the threshold of consumer preference, occasionally rendering these oils unpalatable or commercially unviable as standalone products. To mitigate this issue, traditional industrial practices have long relied on conservative physical mitigation strategies, primarily involving the strategic blending of these highly bitter lots with milder, polyphenol-deficient EVOOs. In these scenarios, the bitter oil serves effectively as a natural antioxidant reservoir to enhance the stability of the final blend. However, this approach merely dilutes the compounds rather than transforming them. To avoid the production of olive oil with an unwanted bitter taste, studies have been carried out, including the immersion of olives in hot water before oil extraction [51] or oil washing with a liquid–liquid phenol extraction procedure in water [52]. Washing with water to reduce bitterness unfortunately also indiscriminately reduces a substantial fraction of desirable phenolic compounds from olive oil. Conversely, targeted biotechnological approaches leveraging the metabolic potential of endogenous, oil-borne oleuropeinolytic yeasts offer a sustainable, chemical-free paradigm to modulate sensory profiles, converting otherwise unmarketable or unpalatable batches into high-value commercial oils. A pioneering strategy in this domain involves the implementation of controlled biological blending, as demonstrated in a comprehensive study by Zullo et al. [53]. This biotechnological approach entails mixing a highly bitter monovarietal Don Carlo EVOO with a milder monovarietal Leccino EVOO, the latter acting as a natural vehicle rich in active, indigenous oleuropeinolytic yeast strains. In detail, from the batch of freshly produced veiled oil twenty-four hermetically closed commercial metallic canisters containing 5 L of EVOO were prepared. Tested conditions were as follows: (1) Don Carlo EVOO blended with 20% settled Leccino EVOO (8 canisters); (2) Don Carlo EVOO blended with 20% decanter Leccino EVOO (8 canisters); (3) Pure Don Carlo EVOO (8 canisters, control). Each treatment group, including the untreated control, was sub-divided into two groups: the first group was stored at 15–16 °C in a fixed position for 3 months, while the second was stored for 3 months under the same conditions, but the canisters were inverted every 7 days. A manual rotary forklift (LLOLDI, Reggio Emilia, Italy) suitable for simultaneously inverting four canisters was used to impede sedimentation of the suspended materials. The resident yeast biota synthesizes and releases extracellular and intracellular β-glucosidases and esterases into the aqueous microphase of the oil emulsion. These biocatalysts systematically cleave the glycosidic bonds of the bitter secoiridoids. Specifically, β-glucosidase and esterase hydrolyzes oleuropein and its aglycones into elenolic acid and hydroxytyrosol, both of which possess a non-bitter taste (Figure 2). Crucially, throughout this bio-debittering kinetic window, all core physicochemical, quality, and purity metrics, such as free fatty acidity, peroxide value, and spectrophotometric ultra-violet indices (K_232_, K_270_, and ∆k) remain strictly within the stringent statutory limits designated by European Union regulations [50] for the premium “Extra Virgin” commercial class (Table 3). Although this biological approach necessitates a regulatory trade-off, as the resulting oil legally loses its single-cultivar designation and must be marketed as an EVOO blend, it represents an innovative and sustainable pathway to rebalance complex organoleptic profiles. To bypass the legal limitations associated with losing monovarietal status on the commercial label, an alternative biotechnological strategy exploits natural, selective decantation during bulk storage. This process is highly effective for monocultivar Coratina EVOO, which is renowned for intensely bitter and pungent organoleptic traits. Research conducted by Ciafardini and Zullo [54] established that storing freshly extracted, unfiltered Coratina oil in 200-L industrial stainless steel tanks at 15–16 °C for a duration of 3 months induces a significant, self-limiting biological debittering phenomenon. Freshly produced EVOO naturally exists as a transient emulsion where a minor fraction of vegetation water and olive pulp particles remains suspended within the continuous triacylglycerol matrix. This specialized aqueous microphase serves as an ideal ecological niche, concentrating both the target secoiridoid substrates and the endogenous oleuropeinolytic yeast cells. As these specialized yeasts proliferate and metabolize the suspended microdroplets, their hydrolytic enzymes progressively reduce the perceived sensory note of bitterness by 33%. This debittering effect was confirmed across independent sensory panel replicates with a statistically significant reduction (*p* < 0.05) compared to the freshly extracted oil [54]. Following this 3-month sediment-contact phase, the oil is subjected to standard industrial filtration to remove the settled yeast biomass and residual water, effectively stabilizing the matrix and locking in the optimized sensory attributes (Table 3). Because no external oils or additives are introduced, the final product fully maintains its highly marketable status as a monocultivar Coratina EVOO, while displaying a balanced, consumer-friendly sensory profile that falls entirely within the regulatory thresholds of the commercial EVOO class [50]. The industrial scalability of these biotechnological interventions remains heavily dependent on precise process monitoring, particularly regarding physical parameter boundaries. The success of both biological blending and selective decantation hinges on maintaining a strict equilibrium with the biotic-abiotic cross-talk. While a sufficient water content (above 0.25% *w/w*) is thermodynamically necessary to maintain yeast viability and enzymatic functionality, an excess of moisture or prolonged sediment exposure can trigger a detrimental metabolic shift. Therefore, the future integration of these indigenous microbial tools into mainstream olive oil downstream processing demands advanced engineering solutions, including the implementation of non-destructive, real-time sensors to track water activity and vertical temperature gradients within large-scale industrial silos. To guarantee the safety and predictability of these non-additive biotechnological interventions at an industrial scale, producers must implement strict risk mitigation frameworks. Since Eu regulations strictly prohibit the use of exogenous starter cultures or chemical preservatives, risk management relies entirely on preventive screening and process control. First, raw material sorting represents the primary quality gate; olive lots affected by advanced bruising or olive fruit fly (*Bactrocera oleae*) attacks must be excluded from long-term decantation, as they are heavily colonized by generalist spoilage yeasts like *B. acidodurans*. Second, rapid screening of the freshly extracted veiled oil using high-throughput molecular tools (such as qPCR assays targeting specific lipolytic markers) or rapid fluorometric lipase activity tests can provide an early microbial profile within hours. If a batch displays a high initial density of dangerous lipolytic species like *C. adriatica*, the spontaneous decantation strategy must be aborted. Instead, the producer must immediately route the batch to high-efficiency cellulose press filtration. As established in Figure 1, reducing the moisture baseline down to 0.06% fundamentally disrupts the yeast cell membrane stability and halts all potential spoilage kinetics within 30 days. Conversely, for compliant batches undergoing decantation, real-time volatile monitoring (e.g., tracking markers like 4-ethylphenol or short-chain fatty acids) allows producers to determine the exact kinetic tipping point, enabling timely racking and filtration to separate the sediment before sensory defects emerge.

## 5. Concluding Remarks, Research Gaps, and Limitations

The study of VOO yeast biota has transitioned from basic isolation to targeted technological application, yet several critical limitations and research gaps remain to be addressed before industrial scaling can be fully realized.

***Methodological limitations:*** A major limitation in current VOO microbiology is the historical reliance on culture-dependent isolation techniques. Although D1/D2 26S rDNA sequencing has successfully identified 27 distinct species, culture-dependent workflows inherently overlook the viable but non-culturable (VBNC) microbial fractions. These silent populations might play unquantified roles in lipid degradation or volatile compound alteration during long-term storage.

***Identified research gaps:*** Firstly, the precise molecular triggers that regulate the synthesis of extracellular lipases versus β-glucosidases in dimorphic yeasts remain largely unknown. Secondly, while blending and decantation have proven effective at a laboratory or small pilot scale, there is a distinct lack of mathematical models to predict yeast kinetic degradation or survival when scaling up to industrial stainless steel silos exceeding 10,000 L. Lastly, the interaction between the dominant yeast biota and the low-abundance bacterial taxa (such as *Pantoea* and *Pseudomonas* species) represents a completely unexplored field of study that could impact overall oil stability.

***Future perspectives:*** Future research must prioritize the integration of multi-omics approaches (metagenomics, metatranscriptomics, and volatilomics) to map the functional mechanics of the oil-borne microbiota in real-time. To make these insights actionable for the industry, immediate research priorities must focus on scaling up validation trials in large-scale industrial silos exceeding 10,000 L. Developing non-destructive, sensor-based monitoring tools must specifically target the real-time tracking of water activity (a_w_) above the critical 0.25% *w*/*w* threshold and the control of vertical temperature gradients (ideally maintained at 15–16 °C). Monitoring these specific parameters first will allow producers to precisely identify the kinetic tipping point where resident yeasts shift from beneficial debittering biocatalysts to active spoilage agents, allowing timely intervation. Ultimately, unlocking the genomic potential of these indigenous, niche-adapted yeasts will provide the olive oil industry with biocatalytic solutions aligned with strict international regulatory frameworks, optimizing both sensory premium quality and commercial longevity.

## Figures and Tables

**Table 1:** Yeast species isolated from virgin olive oil and by-products and identified by D1/D2 rDNA sequencing.

Yeast Species	Substrate	Country	Reference
*Brettanomyces acidodurans* ^1^	Virgin olive oil, spoiled olive oil	Spain, Israel	[17]
*Brettanomyces californica*	Virgin olive oil	Italy	[5]
*Candida adriatica* ^1^	Olive oil sediment, virgin olive oil, spoiled olive oil, oil from decanter	Slovenia, Italy, Croatia	[13,18]
*Candida dendronema*	Virgin olive oil	Italy	[19]
*Candida norvegica*	Virgin olive oil	Italy	[15]
*Candida oleophila*	Virgin olive oil	Italy	[15]
*Candida parapsilosis*	Virgin olive oil	Italy	[20]
*Candida temnochilae*	Virgin olive oil	Italy	[19]
*Debaryomyces hansenii*	Virgin olive oil	Italy	[15]
*Groenewaldozyma auringiensis*	Virgin olive oil	Spain	[17]
*Kuraishia capsulata*	Virgin olive oil	Italy	[5]
*Kuraishia mediterranea* ^1^	Olive oil sediment, spoiled olive oil, virgin olive oil	Slovenia, Portugal, Italy	[21]
*Lachancea fermentati*	Virgin olive oil, olive oil must ^2^	Spain, Italy	[17,22]
*Meyerozyma guilliermondii*	Virgin olive oil	Italy	[23]
*Nakazawaea molendinolei* ^1^	Virgin olive oil, spoiled olive oil, oil from decanter, olive oil must ^2^	Italy, Croatia, Slovenia, Israel	[13,18,22]
*Nakazawaea wickerhamii* (syn. *Candida wickerhamii*)	Virgin olive oil, oil from decander, olive oil must ^2^	Italy	[13,20,22]
*Novakomyces olei* ^1^	Virgin olive oil	Spain	[24]
*Ogataea histrianica* ^1^	Olive oil sediment, virgin olive oil	Slovenia, Italy	[25]
*Ogataea kolombanensis* ^1^	Olive oil sediment, virgin olive oil	Slovenia, Italy	[25]
*Rasporella oleae* ^1^	Olive oil sediment	Slovenia	[26]
*Saccharomyces cerevisiae*	Virgin olive oil	Italy	[19]
*Wickerhamomyces anomalus*	Virgin olive oil	Italy	[15]
*Yamadazyma diddensiae* (syn. *Candida diddensiae*)	Virgin olive oil	Italy	[23]
*Yamadazyma mexicana*	Virgin olive oil	Italy	[19]
*Yamadazyma nakazawaea*	Virgin olive oil	Italy	[19]
*Yamadazyma oleae* ^1^	Virgin olive oil	Italy	[27]
*Yamadazyma terventina* ^1^	Virgin olive oil, oil from decanter	Italy	[13,28]

^1^ Species described as new from olive oil environments. ^2^ Olive oil must: aqueous phase obtained during olive oil extraction before final separation.

**Table 2:** Specific impact of oil-borne yeast group on volatile organic compounds (VOCs) and associated sensory defects in extra virgin olive oil (EVOO) during storage.

Yeast Group	Primary Enzymatic Activity	Key Volatile Markers Accumulation (VOCs)	Depleted Desirable Volatile Compounds	Resulting Sensory Defect (IOC Classification)	Reference
*Brettanomyces* (e.g., *B. acidodurans*)	● Extracellular lipase ● Hydroxycinnamic acid decarboxylase	● 4-ethylphenol ● Acetic acid ● Ethyl acetate	● Hexanal ● *trans*-2- hexenal	● Phenolic medicinal ● Winey-vinegary ● Fusty/muddy	[17,29]
*Candida* (e.g., *C. adriatica*)	● High-affinity glycerol-ester hydrolase	● Free fatty acids (FFA) ● 2-Octanone ● Saturated aldehydes	● *trans*-2- hexen-1-ol ● *cis*-3- hexenyl acetate	● Rancid ● Muddy/sediment	[12,13,34]
*Nakazawaea* (e.g., *N*. *wickerhamii*)	● Esterase ● Dimorphic metabolic pathways	● *trans*-2- heptenal ● 6-Methyl-5- hepten-2-one ● Butanoic acid	● C6 volatile carbonyls ● Hexyl acetate	● Muddy-sediment ● Fusty	[13,34]
*Yamadazyma* (e.g., *Y. terventina*)	● Lipolytic and oxidative enzymes	● 6-Methyl-5- hepten-2-one ● Short-chain fatty acids	● Total positive fruitiness esters	● Rancid ● Off-flavor masking	[13,45]
Pectinolytic sediment yeasts (e.g., *K. mediterranea*, *O. histrianica*)	● Polygalacturonase ● Methanol assimilation	● Methanol ● Galacturonic acid derivatives	● Complex matrix volatile esters	● Fust/muddy sediment degradation	[21,25,31]

IOC, International Olive Council.

**Table 3:** Biotechnologies for debittering highly bitter extra virgin olive oil (EVOO) during storage.

Blending of monocultivar Don Carlo EVOO, characterised by a very bitter taste, with monocultivar Leccino EVOO rich in oleuropeinolytic yeasts and storage in a dark place at 15–16 °C for 3 months	● Debittering of Don Carlo EVOO ● All analytical results remained within parameters established by EU regulations for commercial EVOO class	[50,53]
Decantation of monocultivar Coratina EVOO, characterised by a very bitter taste, in 200-L stainless steel tanks at 15–16 °C for 3 months	● The sensorial note of bitterness was reduced by 33% without altering other qualitative parameters ● Values fell within limits of the commercial EVOO class	[50,54]

## Data Availability

No new data were created or analyzed in this study. Data sharing is not applicable to this article.
